# MicroRNA-488 regulates zinc transporter SLC39A8/ZIP8 during pathogenesis of osteoarthritis

**DOI:** 10.1186/1423-0127-20-31

**Published:** 2013-05-20

**Authors:** Jinsoo Song, Dongkyun Kim, Chang Hoon Lee, Myeung Su Lee, Churl-Hong Chun, Eun-Jung Jin

**Affiliations:** 1 Department of Biological Sciences, College of Natural Sciences, Wonkwang University, Iksan, Chunbuk 570-749, South Korea; 2 Department of Internal Medicine, Division Rheumatology, Wonkwang University School of Medicine, Iksan 570-749, South Korea; 3 Department of Orthopedic Surgery, Wonkwang University School of Medicine, Iksan, Chunbuk 570-749, South Korea

**Keywords:** Osteoarthritis, miR-488, ZIP8, Human articular chondrocytes, Cartilage

## Abstract

**Background:**

Even though osteoarthritis (OA) is the most common musculoskeletal dysfunction, there are no effective pharmacological treatments to treat OA due to lack of understanding in OA pathology. To better understand the mechanism in OA pathogenesis and investigate its effective target, we analyzed miRNA profiles during OA pathogenesis and verify the role and its functional targets of miR-488.

**Results:**

Human articular chondrocytes were obtained from cartilage of OA patients undergoing knee replacement surgery and biopsy samples of normal cartilage and the expression profile of miRNA was analyzed. From expression profile, most potent miR was selected and its target and functional role in OA pathogenesis were investigated using target validation system and OA animal model system. Among miRNAs tested, miR-488 was significantly decreased in OA chondrocytes Furthermore, we found that exposure of IL-1β was also suppressed whereas exposure of TGF-β3 induced the induction of miR-488 in human articular chondrocytes isolated from biopsy samples of normal cartilages. Target validation study showed that miR-488 targets ZIP8 and suppression of ZIP8 in OA animal model showed the reduced cartilage degradation. Target validation study showed that miR-488 targets ZIP8 and suppression of ZIP8 in OA animal model showed the reduced cartilage degradation.

**Conclusions:**

miR-488 acts as a positive role for chondrocyte differentiation/cartilage development by inhibiting MMP-13 activity through targeting ZIP-8.

## Background

Osteoarthritis (OA) is the most common musculoskeletal disorder and the most prevalent articular pathology induced by multiple factors such as obesity, anatomic abnormalities, joint instability, and joint injury [1,2]. OA is characterized by degradation of extracellular matrix macromolecules and decreased expression of chondrocyte protein and resulted severe joint pain, loss of movement, and progressive irreversible dysfunction [3,4]. Epidemiological studies showed that many factors including endogenous as well as exogenous risk factors, could contribute OA pathology directly or indirectly [5,6]. Currently, there are no effective pharmacological treatments to treat OA although some drugs reduce symptoms and slow the progression of OA. Further investigation and understanding of OA pathology is needed and important to develop effective therapeutic targets to control OA.

MicroRNAs (miRNA) are single-stranded RNA of 18–24 nucleotides generated by sequential processing of long RNA transcripts by two key RNase III proteins, Drosha and Dicer [7]. They bind to 3’ untranslated region of target messenger RNAs and either cleavage the mRNAs or repress translation depending on perfect pairing/imperfect pairing [8,9]. Although some algorithms are used to predict potential mRNA targets, only a few miRNAs have been validated and assigned to specific mRNAs. A select number of miRNAs have been shown to play key roles in diverse regulatory pathways, including control of development [10,11], cell proliferation/differentiation [12,13] and many other physiological or pathological processes [14,15]. Studies on Dicer-null mice showed a greatly decreased chondrocyte proliferation and accelerated hypertrophy leading to severe growth defects and premature death of mice indicating the important role of miRNAs in cartilage function [16]. A specific miRNA has been noted not only as key molecules in intracellular regulatory networks, but also as biomarkers for various pathological conditions [17]. Recently, specific miRNAs were reported to be involved in chondrogenesis and inflammatory cartilage diseases [18,19]. MiR-675 regulates type II collagen in articular chondrocytes [20], miR-18 regulates chodnrocytic phenotype by targeting Ccn2/Ctgf [21]. Despite considerable evidences regarding the involvement of miRNAs in cartilage development [22,23], identifications and functions of miRNAs in cartilage development/degeneration are poorly understood.

In the present study, to better understand the molecular mechanisms involved in the OA pathology, we identify miRNAs from normal and OA chondrocytes and characterize the functional role of miRNA-488, which could have important diagnostic and therapeutic potential.

## Methods

### Primary cultures of human chondrocytes

Human chondrocytes were prepared from macroscopically severely damaged zones of osteoarthritic knee joints obtained undergoing total knee replacement or biopsy of normal cartilages. The study was carried out in full accordance with Wonkwang University ethics guidelines and cartilage samples were collected after obtaining written informed consent of the donors. Cartilage small slices were sequentially digested with 0.06% collagenase (Sigma) then seeded at a density of 1.5 × 10^4^ cells/cm^2^ in culture medium consisting of DMEM (Gibco-Invitrogen) supplemented with 10% fetal bovine serum (FBS), 100 IU/ml penicillin, and 100 μg/ml streptomycin (Gibco-Invitrogen).

### Real-time quantitative reverse transcription-polymerase chain reaction (RT-PCR)

The PCR program consisted of an initial denaturation step at 95 °C for 10 min, followed by 40 cycles of 10 sec at 95 °C, 15 sec at 60 °C and 17 sec at 72 °C using to amplify human type II collagen (5’-tgtacgtgaacctgctattgccct-3’ and 5’-taccacgtgcatgtgaaagggact-3’), human MMP-13 (5′-ttgcagagcgctacctgagatcat-3′ and 5′-tttgccagt cacctctaagccgaa-3′) human ZIP1 (5’-gctaaccatg aaggctcagctt-3’ and 5’-ccccgcgaaacagcttacta-3’), human ZIP2 (5’- catcaccg gctagtcctcaga-3’ and 5’-aaccctgctcccaggaaaac-3’), human ZIP3 (5’-tccctgctccccgtgaa-3’ and 5’-cgagcgatgggccttct-3’), human ZIP4 (5’-gtgtgtgggacacggtatgc-3’ and 5’-tgttccgacagtccata tgca-3’), human ZIP6 (5’-g caggctgtcctttataatgca-3’ and 5’-aattcctgttgccattccaaga-3’), human ZIP7 (5’-ctacttcagatcttgctc agttttgc-3’ and 5’-tgaggtg caggaaagcatctc-3’), human ZIP8 (5’-gatcggcccaagcacaaa-3’ and 5’- acaggaatccatatccccaaact-3’), human ZIP9 (5’-tggcttagagcggaatcga-3’ and 5’- ggtgctgccaatgca aaga-3’), human ZIP10 (5’-caccacggcgagaacaaaa-3’ and 5’- cttgtggtgccactggtgat-3’), human ZIP12 (5’-agcagaagccgtgggagtt-3’ and 5’- tggtcaccagcagagagaacct-3’), using monitoring SYBR Green I. To normalize the output, the expression of each gene of interest was divided by GAPDH gene (5'-gatcatcagcaatgcctcct-3' and 5'-tgtggtcatgagtccttcca-3') expression, a commonly used housekeeping gene.

### RNA preparation and miRNA real-time PCR

Total RNA was isolated using the mirVana miRNA isolation kit (Ambion). miRNA analysis was performed using RT^2^ miRNA PCR Arrays (Qiagen) and individual miRNA expression were independently quantified using the TaqMan MicroRNA (Applied Biosystems), according to the manufacturer’s protocols.

### Production of lentiviral particles

The hsa-miR-488 and Negative Control lentivirus was transfected with 3rd generation packaging mix from Applied Biological Materials Inc. (ABM) into human 293FT cells using Lentifectin(ABM) in Opti-MEM I medium (Invitrogen, CA) and cultured overnight. The supernatant was collected and lentiviral particles were concentrated using Lenti-X Concentrator (Clontech, CA).

### Experimental OA and histology of OA cartilage

Experimental OA was induced by destabilization of the medial meniscus (DMM) surgery 8-week-old male mice. Sham-operated animals injected with empty lentiviruses (mock transduction) were used as controls for DMM. Mice were killed 8 weeks after DMM surgery or 2 weeks after intraarticular injection (1 × 10^9^ plaque-forming units (PFU)) of si-ZIP-8-expressing lentiviruses for histological and biochemical analyses. Cartilage destruction in mice was examined using Safranin O staining. Briefly, knee joints were fixed in 4% paraformaldehyde, decalcified in 0.5 M EDTA (pH 7.4) for 14 days at 4 °C, and embedded in paraffin. The paraffin blocks were sectioned at 6 μm thickness. The sections were deparaffinized in xylene, hydrated with graded ethanol, and stained with Safranin O.

### Actin staining

Cells grown on coverslips were washed three times with phosphate-buffered saline (PBS) and then fixed with 4% paraformaldehyde in PBS for 10 min, washed three times with PBS, permeabilized with 0.1% Triton X-100 in PBS for 5 min at room temperature. After washing three times in PBS, cells were blocked with 1% bovine serum albumin (BSA) for 1 h at room temperature. Incubation with Alexa fluor 488-conjugated phalloidin (Invitrogen) was performed in blocking solution (1% BSA in PBS) for 1 hour at room temperature in a light-proof box. Nuclei were stained with 4,6-diamidino-2-phenylindole (DAPI, Santa Cruz Biotechnologies). Samples were mounted in mounting medium (Biomedia).

## Results and discussion

**Figure 1:**
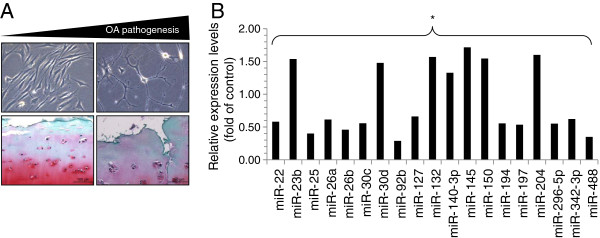
**Expression profile of miRNAs in OA chondrocytes.** (**A**) Images of human articular chondrocytes isolated form OA cartilages (upper panel) and safranin O staining of OA cartilage (lower panel). (**B**) Changes in miRNA level were determined by real-time PCR. *, statistically different from control cells (P < 0.005).

As previously mentioned, miRNA has been shown to involve in various pathological conditions [18-23]. To identify miRNA involved in osteoarthritic conditions, 10 osteoarthritic (OA) cartilages were obtained from patients diagnosed with OA according to the American College of Rheumatology (ACR) criteria, which underwent joint surgery and 5 normal cartilages were obtained from biopsy sample of normal patients. OA cartilage was confirmed by a degenerative morphology with OA progression and staining with Safranin O (Figure 1A). Articular chondrocytes were isolated, cultured, and the expression levels of miRNAs using RT^2^ miRNA PCR Arrays kit. Among miRNA analyzed, miR-23b, miR-30d, miR-132, miR-140-3p, miR-145, miR-150, miR-204 were up-regulated in OA chondrocytes whereas miR-22, miR-25, miR-26, miR-30c, miR-92b, miR-127, miR-194, miR-197, miR-296-5p, miR-342-3p, miR-488 were down-regulated in OA chondrocytes (Figure 1B). Particularly, miR-488 was decreased more than 60% in OA chondrocytes compared to normal chondrocytes suggesting its positive role in articular chondrocytes.

**Figure 2:**
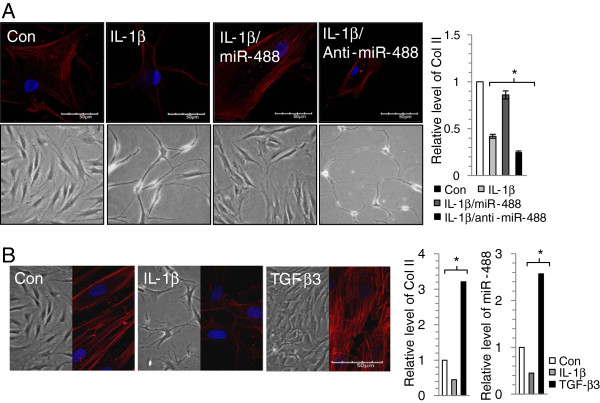
**MiR-488 is involved in degeneration of human articular chondrocytes.** (**A**) Human articular chondrocytes isolated from autopsy normal cartilage were treated with 10 ng/ml IL-1β in the presence of miR-488 or miR-488 inhibitor (anti-miR-488), stained with phalloidin (left panel), and expression level of type II collagen was analyzed (right panel). (**B**) Human articular chondrocytes isolated from autopsy normal cartilage were treated with 10 ng/ml IL-1β or 5 ng/ml TGF-β3, stained with phalloidin (left panel), and expression level of type II collagen and miR-488 were analyzed by real-time PCR (right panel). *, statistically different from control cells (p < 0.001). The error bars represent average of data from each human sample.

The processing of precursor (pre) miR 488* stem-loop produces two mature miRNAs, namely miR 488* and miR 488. Pre miR 488 was almost 90% conserved among human, mouse, rat, cow and horse genomes and the mature miR 488 was found to be 95% conserved overall and 100% identical in the seed region. However, the expression of miR-488 in normal human tissues and disease states has not been extensively studied. To validate the role of miR-488 in human articular chondrocytes, chondrocytes isolated from biopsy cartilage of normal patients were treated with IL-1β. Normal chondrocytes treated with IL-1β displayed degenerative characteristics, i.e. degenerative morphology, inhibited cytoskeletal reorganization as assayed by phalloidin staining, suppressed level of type II collagen RNA as assayed by real-time PCR. However, this degenerative characteristics induced by IL-1β was recovered by co-introduction of miR-488 precursor (Figure 2A). Most significant degeneration was occurred with co-induction of miR-488 inhibitor. Furthermore, exposure of cells to IL-1β, a factor induced degeneration of articular chondrocytes [24] as confirmed by suppression of type II collagen level, down-regulated miR-488 level whereas exposure of cells to TGF-β3, a factor induced differentiation/proliferation of articular chondrocytes [25,26] as confirmed by stimulation of type II collagen level, up-regulated miR-488 level (Figure 2B).

Proteolytic degradation of cartilage by matrix-degrading enzymes such as collagenase 3 (matrix metalloproteinase 13, MMP-13) is a hallmark of osteoarthritis (OA). It has been shown that IL-1β induced MMP-13 during cartilage degradation in OA joints [27]. Consistent with previous reports, the RNA level MMP-13 was increased with IL-1β treatment in a dose dependant manner (Figure 1A left panel). Treatment of 10 ng/ml IL-1β increased the MMP-13 RNA level in a time dependant manner and also induced protein and activation level of MMP-13 (Figure 1A right panel).

**Figure 3:**
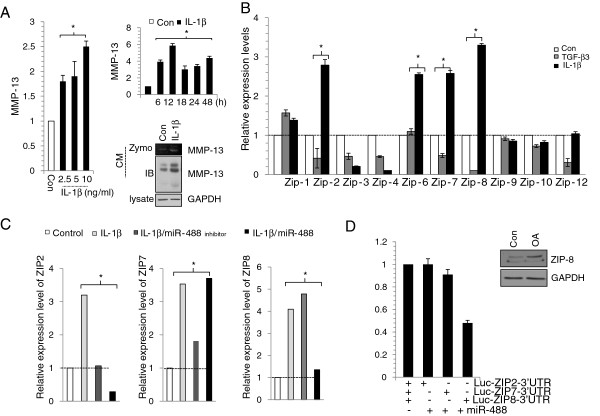
**MiR-488 targets ZIP8. Human articular chondrocytes isolated from autopsy normal cartilage were isolated as described in Material and Method.** (**A**) Articular chondrocytes were treated 2.5, 5, or 10 ng/ml IL-1β and expression level of MMP-13 was examined by real-time PCR (left panel). Cells were treated with 10 ng/ml of IL-1β, expression level was analyzed at indicated time points by real-time PCR (right upper panel), and activation of MMP-13 was analyzed and zymography (Zymo, right lower panel) and immunoblotting (IB, right lower panel), respectively. (**B**) Expression levels of ZIPs were analyzed by real-time PCR. (**C**) Human articular chondrocytes isolated from autopsy normal cartilage were treated with 10 ng/ml IL-1β in the presence of miR-488 or miR-488 inhibitor (anti-miR-488) and expression levels of ZIP2, -7, -8 were examined by real-time PCR. (**D**) Luciferase reporter gene assays of cells expressing the construct containing the human ZIP2, -7, -8 3’-UTR in the absence or presence of miR-488. The expression level of ZIP-8 was analyzed by immunoblotting in normal and OA chondrocytes.

Several recent studies demonstrate the correlation between miRNAs and MMP-13 in human OA chondrocytes [28-31]. It has been known that miR-140 [28] and miR-27 [30] negatively regulates MMP-13 expression indirectly by modulating NF-κB signaling or targeting BMP-7 and miR-27b binds directly with the 3’UTR of human MMP-13 mRNA [31]. In this study, miR-488 did not directly bind to 3’ UTR of MMP-13 in articular chondrocytes (data not shown) suggesting indirect regulation of MMP-13 by miR-488. Since Zn^2+^ is required for catalytic activity of MMP-13 [32], we next asked if miR-488 create the local environment for MMP-13 activation through modulation of Zn^2+^ concentration. Zn^2+^ concentrations are high in bone, cartilage, and teeth and bone growth retardation has been reported in Zn^2+^-deficient conditions [33] indicating Zn^2+^ may play a role in bone/cartilage metabolism. Homeostasis of Zn^2+^ is tightly controlled by two major families of Zn transporters, Zn importers (SLC39s/ZIPs) and exporters (SLC30s/ZnTs) [34]. Among the ZIP family of metal-ion transporters, ZIP-2, ZIP-6, ZIP-7, and ZIP-8 was increased with exposure of human articular chondrocyte to IL-1β and ZIP-2, ZIP-7, and ZIP-8 were decreased with exposure of cells to TGF-β3 (Figure 3B). ZIP-2, ZIP-7 and ZIP-8 were conversely regulated by IL-1β and TGF-β3. Particularly, ZIP-8 showed 300% increase by IL-1β and 90% decrease by TGF-β3. Previous report [35] showed that type X collagen, a marker for hypertrophic chondrocytes, was decreased and defected in the maturation of chondrocytes in Slc39a13-KO mice.

We next asked if the observed induction of MMP-13 activity by IL-1β is due to the modulation and interaction of miR488 to ZIP. Among ZIP-2, -7, -8 whose inductions were reversely regulated by IL-1β and TGF-β3, increased level of ZIP-8 by IL-1β inhibited with co-introduction of miR-488. And most significant increase in ZIP-8 induction was occurred when cells were exposed to miR-488 inhibitor with IL-1β (Figure 3C). To address the direct interaction between miR-488 and ZIP-8, we cloned a segment of ZIP-8 3’UTR (NM_001135147.1, 1700bp ~ 4098bp), co-transfected with miR-488 or negative control in human articular chondrocytes, and luciferase activity was measured after 48 hr. As seen in Figure 3D, miR-488 reduced luciferase activity of the ZIP-8 3’ UTR construct by about 30% as compared to that with the negative control. To investigate the expression level of ZIP-8 during pathogenesis, OA chondrocytes were isolated OA cartilage obtained from patient who undertaken total knee replacement (Figure 3D insert). The protein level of ZIP-8 was dramatically increased in OA chondrocytes compared to normal chondrocytes. Normal chondrocytes were isolated from biopsy tissue of normal patients.

**Figure 4:**
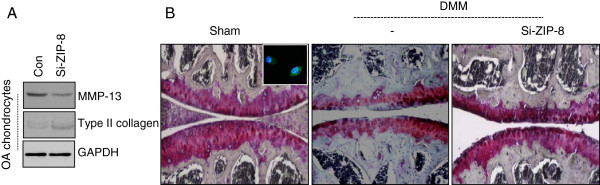
**Suppression of ZIP8 protects DMM-induced cartilage degradation.** (**A**) OA chondrocytes were infected with si-ZIP-8 virus and the expression level of MMP-13, type II collagen was examined by immunoblotting. (**B**) Mouse cartilages with OA induced by destabilization of the medial meniscus (DMM) were stained with Safranin O. Sham-operated (Sham) cartilage was used as control.

Furthermore, the protein level of ZIP-8 was increased in OA chondrocytes compared to normal chondrocytes suggesting the negative role of ZIP-8 during OA pathogenesis (Figure 3D). In OA chondrocytes, the expression level of type II collagen was recovered by the knockdown of ZIP-8 (Figure 4A). In opposition, MMP-13 activation was decreased by the knockdown of ZIP-8. Furthermore, to validate the role of ZIP-8 in cartilage destruction *in vivo*, we suppressed ZIP-8 in cartilage tissue by injecting siZIP-8-expressing lentiviruses into DMM mouse knee joints (Figure 4B). Cartilage destruction caused by DMM surgery was significantly reduced with down-regulation of ZIP-8 suggesting its protective role during OA pathogenesis.

## Conclusion

In sum, our data suggest that miR-488 act as a protective role for chondrocyte differentiation/cartilage development by inhibiting MMP-13 activity through targeting ZIP-8.

## Competing interests

The authors declare that they have no competing interests.

## Authors’ contributions

All authors were involved in drafting the article or revising it critically for important intellectual content, and all authors approved the final version to be published. ML, C-H Chun, and E-JJ had full access to all of the data in the study and take responsibility for the integrity of the data and the accuracy of the data analysis. Study conception and design. ML, C-HC, E-JJ. Acquisition of data. J, DK, JH, CL. Analysis and interpretation of data. JS, DK, JH, CL, ML, C-HC, E-JJ. Manuscript preparation. ML, C-HC, E-JJ.
